# Evaluation of a Biocide Used in the Biological Isolation and Containment Unit of a Veterinary Teaching Hospital

**DOI:** 10.3390/antibiotics10060639

**Published:** 2021-05-27

**Authors:** Catarina Geraldes, Cláudia Verdial, Eva Cunha, Virgílio Almeida, Luís Tavares, Manuela Oliveira, Solange Gil

**Affiliations:** 1 Faculty of Veterinary Medicine, University of Lisbon, Av. Universidade Técnica, 1300-477 Lisboa, Portugal; catarinageraldes96@gmail.com (C.G.); cssverdial@gmail.com (C.V.); evacunha@fmv.ulisboa.pt (E.C.); vsa@fmv.ulisboa.pt (V.A.); ltavares@fmv.ulisboa.pt (L.T.); moliveira@fmv.ulisboa.pt (M.O.); 2 Centre for Interdisciplinary Research in Animal Health (CIISA), Faculty of Veterinary Medicine, University of Lisbon, Av. Universidade Técnica, 1300-477 Lisboa, Portugal; 3 Biological Isolation and Containment Unit (BICU), Teaching Hospital, Faculty of Veterinary Medicine, University of Lisbon, Av. Universidade Técnica, 1300-477 Lisboa, Portugal

**Keywords:** biocide, Virkon™ S, susceptibility, Biological Isolation and Containment Unit, antibiotic

## Abstract

Hospital-acquired infections (HAIs) are a rising problem worldwide, and the best way of coping with them is through infection tracking and surveillance systems, combined with prevention strategies, namely efficient disinfection protocols, that employ various biocides. However, increasing reports about reductions in biocide susceptibility and the development of cross-resistance to antimicrobials emphasize the need for identifying the factors influencing biocide efficiency. In this study, 29 bacterial isolates (*n* = 3 *E. coli*, *n* = 2 *Pseudomonas* spp., *n* = 23 *Enterococcus* spp., and *n* = 1 *Staphylococcus pseudintermedius*), obtained from environmental samples collected from the Biological Isolation and Containment Unit (BICU), of the Veterinary Teaching Hospital of the Faculty of Veterinary Medicine, University of Lisbon, were tested in order to determine their antimicrobial susceptibility to various antibiotics. Thirteen of these isolates were further selected in order to determine their antimicrobial susceptibility to Virkon™ S, with and without the presence of organic matter. Afterward, seven of these isolates were incubated in the presence of sub-lethal concentrations of this formulation and, subsequently, new susceptibility profiles were determined. Fourteen of the 29 isolates (48.3%) were classified as multidrug resistant, all previously identified as enterococci. Concerning Virkon™ S’s susceptibility, the Minimal Bactericidal Concentration (MBC) of this biocide regarding all isolates was at least eight times lower than the concentration regularly used, when no organic matter was present. However, when organic matter was added, MBC values rose up to 23 times. After exposure to sub-lethal concentrations of Virkon™ S, four enterococci presented a phenotypical change regarding antimicrobial susceptibility towards gentamicin. Virkon™ S also resulted in higher MBC values, up to 1.5 times, in the presence of low concentrations of organic matter, but no rise in these values was observed in assays without interfering substance. Virkon™ S seemed to be an efficient formulation in eliminating all bacteria isolates isolated from the BICU. However, organic matter could represent a hindrance to this ability, which emphasizes the importance of sanitization before disinfection procedures. The changes seen in antimicrobial susceptibility could be explained by a general stress-induced response promoted by the sub-lethal levels of Virkon™ S. Additionally, when no organic matter was present, a decrease in susceptibility to this biocide seemed to be non-existent.

## 1. Introduction

Hospital-Acquired Infections (HAIs), also described sometimes as nosocomial infections are a rising problem in veterinary hospitals, as also observed in human hospitals. The increase of these infections in the veterinary field can be attributed especially to the rising number of invasive surgical procedures as well as antimicrobial and immunosuppressive therapies [1]. 

HAIs and are frequently caused by opportunistic pathogens that can be found in either sick or healthy animals [2,3]. These pathogens include bacteria (e.g., methicillin-resistant staphylococci, such as *Staphylococcus aureus* (MRSA) and *S. pseudintermedius* (MRSP), *Escherichia coli*, *Enterococcus* spp., *Salmonella* spp., and *Pseudomonas* spp.), often multidrug-resistant (MDR), viruses (e.g., Influenza, Parvovirus, and Herpesvirus), fungus (mainly *Microsporum canis*) and also parasites (mainly *Cryptosporidium parvum*) [1,2,4]. Several studies have proven the presence of these pathogens on various surfaces (namely the floor, hand contact surfaces, and medical instruments) of veterinary hospitals [5,6,7], which makes these places possible sources of contamination.

It is known that HAIs can lead to dire consequences not only for hospitalized animals, as they are associated with increases in morbidity and mortality rates, but also to the hospitals hosting them (especially financially). Furthermore, considering the zoonotic potential of some of these pathogens, they can also pose a great risk to veterinary personnel and owners [1,4,8].

Although there are no conclusive studies that indicate the proportion of preventable HAIs in veterinary medicine, it is estimated that it is similar to what is established for human medicine [2], which is around 35–55% [9]. This means that the implementation of adequate infection control programs, as well as other measures, could diminish the incidence of these infections in veterinary medicine [1,2,3,10,11,12].

Biocides have been used for a long time with the intent of reducing the number of microorganisms present on different surfaces and are a helpful weapon used for preventing the growing quantity of MDR organisms, the spread of infections, and consequently, the number of HAIs occurring in today’s practice. Regulation (EU) n^o^ 528/2012 of the European Parliament and the Council of 22 May 2012 defines a “biocidal product” as a compound that contains in its composition (or that leads to the formation of) one or more active substances, utilized with the intent of “destroying, deterring or rendering harmless” microorganisms (by other means besides physical or mechanical ones), in order to attenuate or eliminate any detrimental action these agents may have towards host health [13]. These compounds are usually divided into four categories—antiseptics, sterilants, disinfectants, and preservatives, according to their main characteristics and spectrum/mode of action [14,15].

However, it is important to consider that the ability to eradicate different classes of microorganisms presented by each biocide does not solely depend on the type of active substance(s) present. As described by Maillard [16,17], the different factors that can affect biocide efficiency can be divided into three main groups: (i) factors related to the biocide itself, such as concentration, pH, and formulation; (ii) factors related to the environment to which the biocide is applied and how it is applied, such as temperature, presence of organic matter and contact time; and (iii) factors related to the target microorganisms, usually associated with resistance mechanisms such as alterations in biocide penetration (associated with differences in cell wall constitution), the presence of efflux pumps or biofilm formation.

When these factors are not appropriately considered, possible decreases in susceptibility can occur [16,17]. These decreases have been especially concerning due to their possible association with antibiotic resistance. In reports that conclude the existence of this link, common resistance mechanisms (to biocides and antibiotics) such as multidrug efflux pumps [18,19,20,21] and changes in bacterial cell wall permeability [20,22,23] have been reported. 

The biocide tested in this study, Virkon™ S, is a complex formulation with mainly oxidative activity, and its principal constituent is a peroxygen compound named potassium peroxymonosulfate (21.41%). In addition to its main active substance, it is also composed of sodium chloride (1.5%) and other elements (77.09%) such as organic acids, an anionic surfactant, and an inorganic buffer. According to the manufacturer, this formulation is effective against several bacteria, viruses, and fungi [24]. Some recent studies also support this formulation’s high efficiency against a panoply of microorganisms [25,26,27,28,29], but further research is still crucial to better understand the optimum conditions required to maximize its effectiveness. This is critical since some doubts have surfaced regarding its efficiency against higher loads of microorganisms [30]. To the best of our knowledge, there are no studies reporting Virkon™ S’s efficacy against microorganisms present in biofilms. Beyond its broad-spectrum, Virkon™ S is also characterized by being non-corrosive to stainless steel, having a low ecotoxicity/high biodegradability, and low toxicity [31]. However, according to the safety data sheet emitted by Lanxess [32], it can still cause severe eye damage, skin and respiratory irritation, which means safety precautions should always be taken when utilizing this compound.

In the present study, we investigated to which degree certain factors, such as the presence of organic matter, can affect the efficiency of Virkon™ S. We also determined if there is any link between decreased biocide susceptibility and MDR isolates and what influence the presence of a sublethal concentration of this biocide has in the resistance profile of selected bacteria. 

## 2. Results

### 2.1. Antimicrobial Susceptibility Testing

The first step performed in this study aimed to evaluate the antimicrobial susceptibility of the collected bacteria to selected antibiotics in order to establish an initial characterization regarding this group of antimicrobials. This was determined by the disk diffusion method, where isolates were classified as “susceptible”, “intermediate”, and “resistant” according to the zone diameter measurements obtained. Results are presented in Table 1.

When comparing all twenty-nine isolates, the antibiotic responsible for the highest percentage of resistance was tetracycline (89.7%), followed by cefalexin (86.2%), cefotaxime (82.2%), ampicillin (75.9%), amoxicillin-clavulanic acid (65.5%), gentamicin (48.3%), enrofloxacin (44.8%), and ciprofloxacin (27.6%). Trimethoprim-sulfamethoxazole had a resistance rate of 33.3%; however, this percentage excludes the enterococci group due to a specific characteristic of this species. As described, both trimethoprim and sulfamethoxazole molecules act through inhibition of folate synthesis, essential to the production of purines and proteins in the microbial cell. However, *Enterococcus* strains have the ability to extract folic acid from the environment, rendering this mechanism of action obsolete. This ability means that an apparent in vitro susceptibility, obtained when using mediums void of folic acid, may not be equivalent to an in vivo susceptibility [33]; as such, these percentages were not included in Table 1.

The *E. coli* group had a higher susceptibility rate (of 83.3%) to the antibiotics tested when compared to the other groups. The most remarkable result presented by this group was regarding ampicillin susceptibility, being observed that two out of three isolates were classified as resistant, representing the majority of resistances presented by this group.

Regarding the *Pseudomonas* spp. isolates, no breakpoints for ampicillin were found in CLSI [34,35,36], which means no classification was attributed regarding isolates’ susceptibility to this antibiotic. These isolates presented a lower susceptibility rate (of 33.3%) when compared with the previous group. None of the isolates were susceptible to any antibiotic of the β-lactams group (amoxicillin-clavulanic acid, ampicillin, cefalexin, and cefotaxime) or to enrofloxacin or tetracycline, although one isolate did present an intermediate classification to both cefotaxime and enrofloxacin.

The enterococci group had the highest resistance rate (of 74.5%), primarily regarding tetracycline (99.6%) and antibiotics belonging to the β-lactams group (88%).

The single Staphylococcal strain had a susceptibility presented a susceptibility rate of 77.8% and a resistance rate of 22.2%, with resistance only to trimethoprim-sulfamethoxazole and tetracycline.

According to the classification presented by Magiorakos et al. [37], in which an MDR bacteria is defined as a microorganism resistant to one or more antibiotics from three or more antibiotic classes (that vary accordingly to the bacterial species), in this study, fourteen isolates fall into this category, corresponding 48.3% of the total of isolates evaluated, which were all enterococci.

### 2.2. Determination of Virkon™ S’s Minimal Inhibitory Concentrations (MICs) and Minimal Bactericidal Concentrations (MBCs)

After determining the antibiotic susceptibility profile of all isolates, susceptibility to Virkon™ S was also evaluated through the determination of Minimal Inhibitory Concentration (MIC) and Minimal Bactericidal Concentration (MBC) values. These assays were made both in the absence and presence of organic matter through the inclusion of low (low interfering substance) and high (high interfering substance) concentrations of organic matter. This allowed an initial characterization of bacterial susceptibility to Virkon™ S under different environmental conditions. Afterward, the results obtained were also compared to those regarding antibiotic susceptibility in order to determine any possible associations.

Table 2 provides all the mean MIC and MBC values obtained for Virkon™ S.

In assays were performed with no interfering substance (NIS), the MBC of Virkon S was highest in the *E. coli* group (1.250 g/L), followed by the *Pseudomonas* spp. group (0.938 g/L), the enterococci group (0.750 g/L), and finally the staphylococci group (0.625 g/L).

In the assays performed with a low interfering substance (LIS), Virkon™ S’s MBC mean value was highest in the enterococci group (4.911 g/L), followed by the *E. coli* group (2.542 g/L), then the staphylococci (2.500 g/L), and finally the *Pseudomonas* spp. group (1.000 g/L).

Lastly, in assays performed with a high interfering substance (HIS), MBC mean values were considered to be superior to 15.000 g/L among the enterococci group, being important to refer that this was the highest concentration tested. Virkon™ S’s MBC was 14.375 g/L for *S. pseudintermedius*, 12.708 g/L for the *E. coli* group, and 8.750 g/L for the *Pseudomonas* spp. Group.

When compared to MDR isolates, Virkon™ S’s MBC value towards non-MDR isolates was higher in NIS assays (0.775 g/L regarding MDR isolates and 1.063 g/L for non-MDR isolates) but lower in LIS assays (4.750 g/L towards MDR isolates compared to 4.375 g/L for non-MDR isolates).

**Figure 1:**
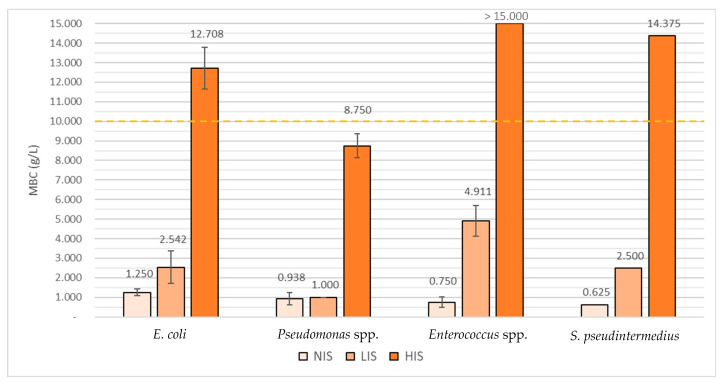
Virkon^®^ S’s of MBCs mean values obtained in the assays with no interfering substance (NIS), low interfering substance (LIS), and high interfering substance (HIS) towards the different groups of isolates (grams per liter). - - - Concentration used in the BICU.

A proportional rise between MBC values and the amount of organic matter used was observed in all the isolates tested, represented in Figure 1.

Virkon™ S’s MBC mean values for the *E. coli* group were approximately 2 times higher in the LIS assays and 10.2 times higher in the HIS assays when compared with the NIS assays. For the *Pseudomonas* spp. group they were, respectively, 1.1 and 9.3 times higher; and for the single *Staphylococcus* isolate, they were 4 and 23 times higher. Virkon™ S’s MBC mean values for the enterococci were 6.5 times higher in the LIS assays; however, the rise regarding the HIS assays could not be determined as it was not possible to establish the exact MBC value (>15.000 g/L). Nevertheless, it is possible to observe that it was at least 20 times higher than the NIS essay. Of major concern are the results corresponding to the Virkon™ S’s MBCs obtained from the HIS assays of the *E. coli* group, the enterococci group, and the Staphylococcal strain since the values obtained surpassed the threshold of 10 g/L, which is the concentration regularly used in the Biological Isolation and Containment Unit (BICU) (Figure 1).

### 2.3. Influence of Bacterial Exposure to Sub-Lethal Concentrations of Virkon™ S

After the establishment of an initial antimicrobial susceptibility profile, the seven enterococci isolates were selected and grown in the presence of a sub-lethal concentration of Virkon™ S. After 24 h of growth, their profiles of antibiotic and Virkon™ S susceptibility were determined.

When comparing the antibiotic susceptibility profiles obtained before and after the induction of bacterial growth at sub-MBC levels (corresponding to half the MBC value obtained in the first determination regarding enterococci isolates), the only alteration noticed was the shift from intermediate to resistant in the susceptibility pattern to gentamicin of four out of seven isolates. These four isolates were the only ones that were not resistant to this antibiotic.

Finally, when comparing Virkon™ S’s MBC values before and after the induction of bacterial growth at sub-MBC levels, a slight decrease in Virkon™ S’s MBCs mean values were noted in the NIS assays (of 0.7 times) and, contradictorily, a significant increase of these values was observed in the LIS assays (of 1.5), as can be seen in Table 3.

As determined before induction of bacterial growth at sub-MBC levels, and compared to Virkon™ S’s MBCs mean values for the MDR isolates, mean values of non-MDR isolates were higher in NIS assays (0.525 g/L for MDR isolates and 0.563 g/L for non-MDR isolates) but lower in LIS assays (7.750 g/L for MDR isolates and 7.188 g/L for non-MDR isolates).

## 3. Discussion

### 3.1. Antimicrobial Susceptibility Testing

Biocides are an indispensable component in the process of reducing and/or eliminating various opportunistic pathogens that are responsible for many negative consequences, such as HAIs. Many of these pathogens are bacteria that have increasingly been classified as MDR, forcing more prolonged treatments and hospital stays [1], but also rendering veterinary hospitals a possible source of transmission for these bacteria to professionals and to the community [6]. This is why the characterization of the bacterial species present in high-risk locations, such as isolation units, as well as the evaluation of their antimicrobial resistance profiles is of utmost importance.

The order of resistance rates presented for each antibiotic tested was especially dictated by the high number of enterococci isolates in this study.

From all the isolates tested, around 48.3% were classified as MDR, all belonging to the enterococci group. High levels of resistance presented by these bacteria are not considered a novelty [33,38,39,40] and have been reported in many studies [41,42], including in bacteria isolated from surfaces in other Veterinary Teaching Hospitals (VTHs) [6]. 

The categorization of the fourteen *Enterococcus* spp. as MDR was made according to the exhibition of a resistant phenotype towards penicillins (ampicillin), fluoroquinolones (ciprofloxacin and enrofloxacin), and tetracyclines (tetracycline), as suggested by Magiorakos et al. [37], since this genus is known to possess intrinsic resistance to cephalosporins and aminoglycosides [33,38,39,40].

The tetracycline resistance rate (99.6%) was higher than the one observed in most of the selected studies [6,43,44,45,46,47,48], with the exception of the works presented by Iseppi et al. [41] and Rodrigues et al. [49], in which similar rates, of 97.5% and 95.2% respectively, were found. This antibiotic is also frequently one to which enterococci have the highest percentage of resistance, along with enrofloxacin [6,41,43,44,45,46,47,48,49]. 

The percentage obtained concerning ampicillin resistance (78.3%) was the second-highest, surpassed only by the values obtained by Ghosh et al. [45] in the United States, of 96.5%. However, this study only focused on *E. faecium* isolates, which are known to usually present a higher level of resistance (i.e., higher MIC values) towards β-lactams than other enterococci species [50].

From all four antibiotics, the resistance rate related to ampicillin was the most worrisome, not only because these results had the greatest differences compared to other studies, but also because β-lactams are still considered the first line of defense against enterococcal infections in both animals (to which amoxicillin is the predominant antibiotic used) [51] and humans (to which ampicillin is the predominant antibiotic used, sometimes combined with an aminoglycoside) [33,38,39].

These higher resistance rates could be explained by contemplating isolate origin. As seen in both studies by Kataoka et al. [46] and Leite-Martins et al. [47], isolates obtained from clinical samples and from animals under antibiotic selective pressure, which possibly represents the majority of cases admitted to the BICU, present a higher resistance rate than isolates collected from healthy animals. This indicates that antimicrobial treatments to different ailments can potentially exert antibiotic pressure on commensal microbiota, leading to the selection of resistant microbial strains [52,53,54,55,56,57,58]. This could be especially true regarding enterococci since they present intrinsic resistance to many of the antibiotics regularly used in these treatments [33,38,39,40,50]. Considering that the majority of the animals admitted to the BICU present some kind of intestinal disease, as stated by Machado et al. [59,60], which commits to alterations in the gut microbiota, the results obtained also become a little more comprehensible. 

These rates become even more worrisome when considering potential zoonotic transmissions of these MDR isolates [61] and also the possibility of the transference of resistance determinants from these bacteria to other species, such as *Staphylococcus aureus* [62,63,64,65]. 

### 3.2. Determination of Virkon™ S’s Minimal Inhibitory Concentrations (MICs) and Minimal Bactericidal Concentrations (MBCs)

Determining MIC and MBC values of different biocide formulations in different functional conditions is an essential practice in order to ascertain how to efficiently eliminate possible HAIs’ agents, such as those tested in this study. The establishment of these values not only enables a better understanding of the optimum conditions of application of the different compounds, consequently assisting in reducing the possible consequences of the interaction between sub-inhibitory/sub-lethal concentrations and different bacteria but also allow for the monitoring of loss of susceptibility to these compounds by bacteria.

As seen in the first assays, when Virkon™ S’s bactericidal concentrations were determined in the absence of organic matter, Gram-negative isolates had a lower susceptibility to this biocide than Gram-positive isolates, which was conveyed by the higher Virkon™ S’s MBC mean values regarding the first group. This corroborates the theory that the lipopolysaccharide barrier that comprises the outer layer of Gram-negative bacteria is responsible for decreasing the activity of many biocides, including membrane-active agents [66], as in Virkon™ S’s case. However, in the presence of organic matter, this tendency was no longer observed.

Beyond the inexistence of an established guideline to test the variability of biocides efficiency, no MIC nor MBC breakpoints are available for these compounds, which makes the categorization of bacteria as resistant or susceptible difficult [67]. This becomes even harder in Virkon™ S’s case since, to the best of our knowledge, no other studies have been published to date regarding MIC and MBC values of this biocide. Nevertheless, considering that the MBC values obtained were approximately eight times lower or more than the concentration regularly used, it seems correct to affirm that, in the absence of organic matter, Virkon™ S is an efficient biocidal agent against all the organisms tested. Even so, it should be considered that these assays were performed using suspended planktonic bacteria, which are usually considered more susceptible to antimicrobials than, for example, bacteria present in biofilm form [68,69].

However, the presence of organic matter is known to alter biocide susceptibility, especially of compounds constituted by oxidizers [70]. Generally, this action seems to be due to three different mechanisms: (i) neutralization of the biocide molecule, reducing its availability; (ii) the formation of a protective barrier around the microorganism; (iii) the formation of microbial aggregates; with (ii) and (iii) being normally associated with biofilm formation [16,71].

According to the results obtained in both LIS and HIS assays, organic matter does seem to reduce Virkon™ S’s bactericidal capacity, especially when applied in high concentrations. In HIS assays, this formulation’s MBC values towards *Enterococcus* spp., *E. coli*, and *Staphylococcus pseudintermedius* surpassed the concentration regularly used in the BICU. Although this was not observed regarding the *Pseudomonas* spp. group there was still a very significant rise in the bactericidal concentration (of nine times the base concentration) towards those isolates. These results suggest that, in the presence of higher loads of organic matter, Virkon™ S may not be effective in eliminating all targeted microorganisms. This is especially problematic when disinfecting cages, which have a high quantity of organic matter, such as urine and or feces, which gives a greater emphasis on the necessity of thorough cleaning procedures before disinfection.

The loss of efficiency presented by this disinfectant in both LIS and HIS assays has also been demonstrated by McCormick and Maheshwari [31] regarding adenovirus and also by Chandler-Bostock and Mellits [72] regarding rotavirus. On the other hand, two studies, one by Wu et al. [29] regarding rabies virus, and another by Skinner et al. [27] regarding *E. coli* O157:H7, indicated that Virkon™ S behaved effectively in the presence of organic matter. This disparity could be explained by the differences in the protocols used, by the different microorganisms tested, and especially by the variations in types of organic matter used, since, as concluded before, this disinfectant could still be efficient in the presence of low quantities of organic matter. No MIC nor MBC values were presented by Skinner et al. [27] concerning Virkon™ S, which makes a comparison between results even more difficult. 

Even though the number of isolates used was low, making it impossible to perform a statistical assessment, the results obtained seem to indicate that there were no associations between decreased biocide susceptibility and MDR isolates. No other studies were found that analyzed this possible correlation concerning Virkon™ S. Nevertheless, other authors performed this same analysis regarding other disinfectants such as Quaternary Ammonium Compounds (QACs), triclosan and other phenolics, ethanol, sodium hypochlorite, hydrogen peroxide, among others, regarding different bacteria. While some authors concluded that there was indeed some degree of correlation between decreased susceptibility to biocides and resistance to antibiotics [73,74,75,76], others concluded the exact opposite [67,77,78,79], sometimes even regarding the same biocide [73,74,77,79]. These different results indicate that this correlation does not only depend upon the active substance tested but also on the target bacteria. However, the significant variations in the protocol should also be considered as a possible explanation for these distinct conclusions.

### 3.3. Influence of Bacterial Exposure to Sub-Lethal Concentrations of Virkon™ S

Upon contact with sub-lethal concentrations of Virkon™ S, a modification of the phenotypic susceptibility pattern from intermediate to resistant was observed in all the isolates that were not gentamicin resistant.

A stress-induced response translates as the capacity that some microorganisms exhibit that allows them to adapt to numerous forms of stress, including chemical stress [80]. This response has been associated with alterations in gene expression and cell physiology which, consequently, can lead to a decrease in antimicrobial susceptibility [81]. This adaptation mechanism could also be indicated as one of the causes of cross-resistance between biocides and antibiotics, especially since both are associated with overexpression of multidrug efflux pumps [18,19,20,21] and changes in cell wall permeability [20,22,23].

On the other hand, *Enterococcus* strains are well-known for their intrinsic aminoglycoside resistance. In gentamicin’s case, the only antibiotic that was associated with a change in susceptibility post bacterial subjection to sub-lethal concentrations of Virkon™ S, this intrinsic resistance is usually associated with poor antibiotic uptake due to cell wall impermeability [33,38,39,40]. 

Taking both of these factors into account, the change in susceptibility profiles observed could be due to an adaptative stress-induced response caused by the presence of the biocide that leads to the alteration in the expression of already existent genes associated with gentamicin resistance.

This could also indicate that this response is not specific to Virkon™ S and could be promoted by similar biocide formulations.

As no increase in Virkon™ S’s MIC and MBC values was observed in the absence of organic matter, it may be an indication that the tested isolates were not able to develop any mechanisms that could lead to a decrease in susceptibility to Virkon™ S. 

However, if this hypothesis were true, it would not explain the rise observed in Virkon™ S’s MBC values when in contact with an interfering substance. The results obtained could mean that organic matter does not only act by neutralization of the biocide, but forms a protective layer around bacteria, promoting their aggregation and consequently reducing their susceptibility to Virkon™ S. The increase seen regarding these MBC values also suggests that there could have been a slight modification to this interaction (between bacteria and organic matter), after bacterial subjection to a subinhibitory concentration of Virkon™ S, either due to enablement in the formation of a protective organic barrier around the microorganism or in the formation of microbial aggregates. As there seem to be few studies concerning the influence of organic matter in bacterial susceptibility to Virkon™ S, further research is still needed in order to better understand this relationship.

## 4. Materials and Methods

### 4.1. Bacterial Isolates

In this study, a collection of 29 bacterial isolates obtained by Verdial [82] were used. These isolates were collected from environmental samples from different surfaces (cages, taps, handles, examination tables, cabinet surfaces, feeding bowls, and sponges) of the BICU at the VTH of the Faculty of Veterinary Medicine, University of Lisbon. These isolates were identified as *Enterococcus* spp. (*n* = 23), *Escherichia coli* (*n* = 3), *Pseudomonas* spp. (*n* = 2), and *Staphylococcus pseudintermedius* (*n* = 1) and kept in cryopreservation tubes.

Four control strains, recommended by the European Standard (EN) 1656 (CEN 2009), were also tested: *Pseudomonas aeruginosa* ATCC 15442, *Staphylococcus aureus* ATCC 6538, *Enterococcus hirae* ATCC 10541, and *Escherichia coli* ATCC 10536.

### 4.2. Antimicrobial Susceptibility Testing

The characterization of the susceptibility profile of all isolates under study was performed through the disk diffusion method, made accordingly to the Clinical and Laboratory Standards Institute (CLSI) guidelines VET01-A4, VET01-S2, and M100S [33,34,35]. A control strain (*E. coli* ATCC 25922) was also tested, as recommended by CLSI, and four independent replicates were performed in order to assure the reproducibility of the results obtained.

The antibiotics to be tested were chosen according to their frequent use as a treatment option in the VTH: amoxicillin-clavulanic acid (AMC), ampicillin (AMP), cefalexin (CL), cefotaxime (CTX), ciprofloxacin (CIP), enrofloxacin (ENR), gentamicin (CN), trimethoprim-sulfamethoxazole (SXT), and tetracycline (TE) (Oxoid Limited^®^, Hampshire, United Kingdom).

Bacterial suspensions with turbidity equivalent to 0.5 in the McFarland scale (1.5 × 10^8^ CFU/mL) were prepared for all isolates. These suspensions were then inoculated using the lawn technique on Mueller–Hinton agar (Oxoid Limited^®^, Hampshire, United Kingdom) plates, followed by placement of the antibiotic disc on the agar surface and after incubation for 18 h at 37 °C, the diameter of the zones of inhibition formed around the disc was measured. Results were interpreted according to the CLSI guidelines VET01-S2 [35] and M100S [36].

### 4.3. Determination of Minimal Inhibitory Concentrations (MICs) and Minimal Bactericidal Concentrations (MBCs)

The MICs and MBCs of the biocide Virkon™ S were determined regarding seven selected enterococci isolates, six obtained from samples collected from areas with higher contact with animals (i.e., examination tables and cages) and one from a sponge located in the preparatory of the BICU; and also for the three *E. coli* isolates, the two *Pseudomonas* spp. isolates and the *S. pseudintermedius* isolates (*n* = 13).

Before each assay, a total of 9 solutions of Virkon™ S (Lanxess^®^, Köln, Germany) were prepared using sterilized water. These volumes represented a range of initial concentrations that, when added into the mix, would represent the final concentrations of 15 g/L, 12.5 g/L, 10 g/L (equivalent to 1%, which is the concentration regularly used in de BICU), 7.5 g/L, 5 g/L, 2.5 g/L, 1 g/L, 0.5 g/L, and 0.25 g/L.

A neutralizer, composed of 3 g/L of sodium thiosulfate (Scharlau^®^, Barcelona, Spain), 30 g/L of polysorbate 80 (AppliChem^®^, Darmstadt, Germany) and 3 g/L of lecithin (Scharlau^®^, Barcelona, Spain), was also prepared in order to neutralize Virkon™ S’s action after a 10-minute contact time. This neutralizer was subjected to an optimization trial and several controls in order to validate the neutralization method and guarantee the absence of toxicity. These controls were performed prior to each assay, and results are presented in supplementary tables (Tables S1 and S2). Both the composition of the neutralizer and control procedures were made according to EN 1656 [83]. 

In order to recreate the organic matter conceivably present in surfaces disinfected with Virkon™ S in the BICU, low-level and high-level interfering substances were also prepared as described in the same European Standard [83]. 

Solutions representative of a low-level interfering substance were prepared by dissolving 3 g of bovine albumin fraction V (AppliChem^®^, Darmstadt, Germany) in 100 mL of water followed by sterilization by membrane filtration using a sterile syringe filter of 0.2 µm (VWR International^®^, Leuven, Belgium). 

The high-level interfering substance solutions were prepared by dissolving 50 g of yeast extract (Scharlau^®^, Barcelona, Spain) in 250 mL of water. The suspension was then sterilized by autoclave and cooled until 20 °C (±1 °C). In a separate container, 5 g of albumin were dissolved in 25 mL of water and then sterilized by membrane filtration using a sterile syringe filter of 0.2 µm, after which 25 mL of yeast extract were added. 

Both solutions were preserved at 8 °C ± 1 °C and used until 1 month after being prepared. Finally, the protocols for MIC and MBC determination were also adapted from EN 1656 [83].

Before the beginning of each assay, three 96-well plates (VWR International^®^, Leuven, Belgium) were prepared. In the first plate (labelled “plate 1”), each well between columns 4 and 12 was filled with 160 µL of Virkon™ S in the different concentrations to be tested, while columns 1 to 3 were left empty.

In the second plate (labelled “plate 2”), all wells between columns 4 and 12 were filled with 160 µL of neutralizer and 20 µL of sterile water. The remaining wells were left empty.

In the third plate (labelled “plate 3”), column 1 was used as a negative control in order to confirm the sterility of the liquid medium used (Trypticase Soy Broth (TSB) (VWR International^®^, Leuven, Belgium)) and filled with 200 µL, while column 3 was used as a positive control in order to confirm the presence of bacteria in the initial bacterial suspension and filled with 180 µL of TSB. Columns 4 to 12 (used for testing) were also filled with 180 µL of TSB, while column 2 was left empty.

After this preparation, and immediately prior to the beginning of each assay, bacterial suspensions with a concentration corresponding to 0.5 McFarland were prepared in the diluent solutions using 24-h cultures in Brain Heart Infusion (BHI) agar (VWR^®^ International, Leuven, Belgium).

All MIC determinations assays were performed at room temperature (20 C (±1 °C)). 

First, all the positive control wells were filled with 20 µL of bacterial suspension. In the assays performed in the presence of interfering substances, 40 µL of a 1:1 mixed suspension of bacteria plus interfering substances (low or high) was added to two consecutive lines of plate 1, containing different concentrations of Virkon™ S.

In assays performed without interfering substances, these were replaced by 20 µL of TSB that was incorporated into the plate before the 20 µL of the bacterial suspension.

After the distribution of all the solutions in plate 1, these were incubated and agitated (at approximately 500 rpm) for a period of 10 min (±10 s) (contact time of Virkon™ S given by the manufacturer) at room temperature. 

Afterward, 20 µL of the suspensions from the wells of plate 1 were transferred to plate 2 (with the 160 µL of neutralizer and 20 µL of sterile water), and this plate was incubated and agitated for 5 min (±10 s) (time taken for the neutralizing substance to act), at room temperature.

Following this step, 20 µL of the suspensions from the wells of plate 2 were transferred to columns 4 to 12 of plate 3 (final plate), filled with 180 µL of TSB. This plate was then incubated at 37 °C (±1 °C) for 24 h. 

The MIC was considered to be the lowest concentration of Virkon™ S that visually inhibited bacterial growth.

In order to determine MBC values, 5 µL were taken from all the wells that showed no bacterial growth and inoculated by spot on lawn into a Trypticase Soy Agar (TSA) (VWR International^®^, Leuven, Belgium) plate. After incubation at 37 °C (±1 °C) for 24 h, these agar plates were observed to detect bacterial colonies’ formation.

### 4.4. Influence of Virkon™ S Sub-MBC Values on Isolates Biocide Resistance Ability

After determination of the MICs and MBCs of Virkon™ S regarding the original isolates, seven *Enterococcus* spp. were selected because this bacterial genus was the one detected in higher numbers in the BICU, and the only group that presented MDR isolates.

Aiming to evaluate the influence of the presence of sub-MBC concentrations of Virkon™ S in the isolates’ resistance profile to this biocide, bacterial suspensions with 0.5 McFarland of the seven selected enterococci were diluted (in a proportion of 1:10) in TSB supplemented with a sub-MBC concentration of Virkon™ S and incubated at 37 °C (±1 °C) for 24 h. After incubation, suspensions were inoculated in TSA, and the isolates obtained after a new incubation at 37 °C for 24 h were used to determine the new MICs and MBCs of Virkon™ S. The antimicrobial resistance profiles of the isolates were also determined towards the antibiotics previously tested using the disk diffusion method, and results were compared to those of the original isolates.

## 5. Conclusions

Virkon™ S appeared to be an efficient compound in eliminating all bacteria tested, which were previously isolated from the BICU; however, further studies should be made in order to evaluate the ability of this compound to remove bacteria in biofilm form. This becomes an even more pressing matter considering the fact that the results obtained seem to indicate that organic matter inactivated Virkon™ S. The results also highlight the importance of cleaning surfaces thoroughly before disinfecting them with this biocide.

After exposure to sublethal concentrations of Virkon™ S, slight alterations were observed in the antimicrobial susceptibility profile of four enterococci regarding gentamicin, possibly due to a chemical stress response caused by this exposure.

Contact with Virkon™ S did not decrease isolate susceptibility to this formulation in the absence of organic matter. However, an increase in MBC values was indeed seen when low levels of organic matter were used. 

Finally, despite not being its main objective, this study emphasizes the rising importance of *Enterococcus* strains as a nosocomial pathogen since it was not only the most frequently isolated bacteria in the BICU, but it was also the isolate that presented a higher frequency of antimicrobial resistance, with a worrisome percentage of MDR isolates (60.9%).

## Figures and Tables

**Table 1:** Results from the antimicrobial susceptibility tests of all isolates under study (*n* = 29), with corresponding susceptibility and resistance rates for each antibiotic tested.

	*E. coli* (*n* = 3)			*Pseudomonas* spp. (*n* = 2)			*Enterococcus* spp. (*n* = 23)			*S. pseudintermedius* (*n* = 1)		
	*n* = S%	*n* = I%	*n* = R%	*n* = S%	*n* = I%	*n* = R%	*n* = S%	*n* = I%	*n* = R%	*n* = S%	*n* = I%	*n* = R%
AMC	100	0	0	0	0	100	26.1	0	73.9	100	0	0
AMP	33.3	0	66.7	-	-	-	21.7	0	78.3	100	0	0
CL	100	0	0	0	0	100	0	0	100	100	0	0
CTX	100	0	0	0	50	50	0	0	100	100	0	0
CIP	100	0	0	100	0	0	0	65.2	34.8	100	0	0
ENR	66.7	0	33.3	0	50	50	0	47.8	52.2	100	0	0
CN	100	0	0	100	0	0	0	38.1	61.9	100	0	0
SXT	100	0	0	50	0	50	-	-	-	0	0	100
TE	33.3	33.3	33.3	0	0	100	0	0.4	99.6	0	0	100

AMC: Amoxicillin-clavulanic acid; AMP: Ampicillin; CL: Cefalexin; CTX: Cefotaxime; CIP: Ciprofloxacin; ENR: Enrofloxacin; CN: Gentamicin; SXT: Trimethoprim-sulfamethoxazole; TE: Tetracycline. *n* = S%: Percentage of isolates susceptible to the antibiotic in question; *n* = I%: Percentage of isolates intermediate to the antibiotic in question; *n* = R%: Percentage of isolates resistant to the antibiotic in question.

**Table 2:** Virkon™ S’s mean MICs and MBCs values towards all isolates tested, divided between assays with no interfering substance (NIS), low interfering substance (LIS), and high interfering substance (HIS) (grams per liter, g/L).

Isolate Code	MDR	MIC (g/L)			MBC (g/L)		
		NIS	LIS	HIS	NIS	LIS	HIS
C1	-	1.000	1.375	11.250	1.000	1.375	11.250
C2	-	1.000	3.125	13.125	1.375	3.125	13.125
C3	-	1.375	3.125	13.750	1.375	3.125	13.750
$\bar{x}$		1.125	2.542	12.708	1.250	2.542	12.708
σ		0.177	0.825	1.062	0.177	0.825	1.062
P1	-	0.875	1.000	9.375	1.250	1.000	9.375
P3	-	0.500	1.000	8.125	0.625	1.000	8.125
$\bar{x}$		0.688	1.000	8.750	0.938	1.000	8.750
σ		0.188	0.000	0.625	0.313	0.000	0.625
E2	-	0.750	5.000	>15.000	0.750	5.625	>15.000
E3	✓	0.500	5.000	>15.000	0.625	5.000	>15.000
E5	✓	1.000	3.125	>15.000	1.375	3.125	>15.000
E14	-	0.625	5.000	>15.000	0.625	5.000	>15.000
E16	✓	0.625	5.000	>15.000	0.750	5.000	>15.000
E17	✓	0.625	5.000	>15.000	0.625	5.000	>15.000
E19	✓	0.500	5.625	>15.000	0.500	5.625	>15.000
$\bar{x}$		0.661	4.821	>15.000	0.750	4.911	>15.000
σ		0.160	0.725	-	0.267	0.778	-
S3	-	0.625	2.500	14.375	0.625	2.500	14.375
$\bar{x}$		0.625	2.500	14.375	0.625	2.500	14.375
σ		-	-	-	-	-	-

C1–C3: *E. coli* isolates; P1 and P3: *Pseudomonas* spp. isolates; E1–E23: *Enterococcus* spp. isolates; S3: *Staphylococcus pseudintermedius* isolate. MDR (✓): Multidrug-Resistant Isolates. $\bar{x}$ : Mean MIC and MBC values of each group; σ: Corresponding standard deviation of the population.

**Table 3:** Virkon™ S’s MICs and MBCs mean values towards the selected *Enterococcus* spp. isolates after induction of bacterial growth at sub-MBC levels, including assays with no interfering substance (NIS), low interfering substance (LIS), and high interfering substance (HIS) (grams per liter, g/L).

Isolate Code	MDR	Assay	MIC (g/L)			MBC (g/L)		
			NIS	LIS	HIS	NIS	LIS	HIS
E2	-	A	0.750	5.000	>15.000	0.750	5.625	>15.000
		B	0.625	7.500	>15.000	0.625	7.500	>15.000
E3	✓	A	0.500	5.000	>15.000	0.650	5.000	>15.000
		B	0.625	6.250	>15.000	0.625	6.250	>15.000
E5	✓	A	1.000	3.125	>15.000	1.375	3.125	>15.000
		B	0.500	6.875	>15.000	0.500	7.500	>15.000
E14	-	A	0.625	5.000	>15.000	0.625	5.000	>15.000
		B	0.500	6.875	14.125	0.500	6.875	14.125
E16	✓	A	0.625	5.000	>15.000	0.750	5.000	>15.000
		B	0.500	8.750	>15.000	0.500	8.750	>15.000
E17	✓	A	0.625	5.000	>15.000	0.625	5.000	>15.000
		B	0.500	8.750	>15.000	0.500	8.750	>15.000
E19	✓	A	0.500	5.625	>15.000	0.500	5.625	>15.000
		B	0.500	7.500	>15.000	0.500	7.500	>15.000
		A	0.661	4.821	>15.000	0.750	4.911	>15.000
		B	0.536	7.500	>15.000	0.536	7.589	>15.000
σ		A	0.160	0.725	-	0.267	0.778	-
		B	0.056	0.884	-	0.056	0.847	-

MDR (✓): Multidrug-Resistant Isolates. $\bar{x}$ : Mean MIC and MBC values of all selected isolates; σ: Corresponding standard deviation of the population. A: Assay performed before the induction of bacterial growth at sub-MBC levels; B: Assay performed after the induction of bacterial growth at sub-MBC levels.

## Data Availability

The data presented in this study are available on request from the corresponding author.

## Supplementary Materials

The following are available online at https://www.mdpi.com/article/10.3390/antibiotics10060639/s1, Table S1. Results obtained, in number of colonies per plate, regarding the neutralizer toxicity controls (assay 1 and assay 2), made in order to guarantee the absence of toxicity by the neutralizer. Different concentrations of sodium thiosulfate (3, 5, 8, 10, 15, 20 g/L were tested). Table S2. Results obtained, in number of colonies per plate, regarding the neutralizing method validation controls (assay 1 and assay 2), made in order to guarantee Virkon S’s neutralization. The number of colonies was determined after a 24- and 48-h period of incubation at 37 °C. Different concentrations of sodium thiosulfate (3, 5, 8, 10, 15, 20 g/L were tested).
