# Supplementary material for: Evaluation of a Biocide Used in the Biological Isolation and Containment Unit of a Veterinary Teaching Hospital
Source: Antibiotics (Basel). 2021 May 27;10(6):639. doi: 10.3390/antibiotics10060639 (PMC8229411; doi:10.3390/antibiotics10060639)
Supplement: Supplementary file 1 [file antibiotics-10-00639-s001.zip › antibiotics-1211821-supplementary.pdf]

Neutralizer Toxicity Control

Table S1. Results obtained, in number of colonies per plate, regarding the neutralizer toxicity controls (assay 1 and assay 2), made in order to guarantee the absence of toxicity by the neutralizer. Different concentrations of sodium thiosulfate (3, 5, 8, 10, 15, 20 g/L were tested).

ATCC Tested	Neutralizer Composition			Number of Colonies Incubation Time (37 °C) 24 hours		48 hours	
				Assay 1	Assay 2	Assay 1	Assay 2
Escherichia coli ATCC 10536	Control			43		43	
	Polysorbate 80 (30 g/L) + Lecithin (3 g/L)	Sodium Thiosulfate (g/L)	3	44	42	44	42
			5	32	40	32	40
			8	34	33	34	34
			10	41	36	41	36
			15	34	44	35	44
			20	43	55	43	55
Enterococcus hirae ATCC 10541	Control			39		39	
	Polysorbate 80 (30 g/L) + Lecithin (3 g/L)	Sodium Thiosulfate (g/L)	3	47	36	47	36
			4	36	38	36	38
			8	39	30	39	30
			10	45	47	45	47
			15	40	28	41	29
			20	38	39	39	39
Pseudomonas aeruginosa ATCC 15442	Control			13		13	
	Polysorbate 80 (30 g/L) + Lecithin (3 g/L)	Sodium Thiosulfate (g/L)	3	10	10	10	11
			5	11	16	13	18
			8	15	16	15	16
			10	10	13	10	15
			15	16	16	17	16
			20	11	14	15	15
Staphylococcus aureus ATCC 6538	Control			35		36	
	Polysorbate 80 (30 g/L) + Lecithin (3 g/L)	Sodium Thiosulfate (g/L)	3	45	40	46	40
			5	32	36	32	36
			8	27	33	27	33
			10	40	33	40	34
			15	43	38	43	39
			20	28	35	28	36

Neutralizing Method Validation

Table S2. Results obtained, in number of colonies per plate, regarding the neutralizing method validation controls (assay 1 and assay 2), made in order to guarantee Virkon S's neutralization. The number of colonies was determined after a 24- and 48-hour period of incubation at 37 °C. Different concentrations of sodium thiosulfate (3, 5, 8, 10, 15, 20 g/L were tested).

Interfering Substance	Neutralizer Composition			Number of Colonies – Incubation Time (37 °C)			
				24 hours		48 hours	
				Assay 1	Assay 2	Assay 1	Assay 2
None	Polysorbate 80 (30 g/L) + Lecithin (3 g/L)	Sodium Thiosulfate (g/L)	3	40	42	42	43
			5	40	47	40	47
			8	52	49	52	49
			10	50	46	51	48
			15	55	51	55	51
			20	49	41	48	41
Low	Polysorbate 80 (30 g/L) + Lecithin (3 g/L)	Sodium Thiosulfate (g/L)	3	74	-	74	-
			5	80	88	80	88
			8	86	70	86	73
			10	92	86	92	88
			15	86	94	88	94
			20	114	105	114	105
High	Polysorbate 80 (30 g/L) + Lecithin (3 g/L)	Sodium Thiosulfate (g/L)	3	172	179	172	179
			5	187	189	191	189
			8	196	191	196	191
			10	189	202	189	202
			15	215	204	215	214
			20	201	209	201	209
